# Advanced *in vitro* Research Models to Study the Role of Endothelial Cells in Solid Organ Transplantation

**DOI:** 10.3389/fimmu.2021.607953

**Published:** 2021-02-10

**Authors:** Daphne M. Peelen, Martin J. Hoogduijn, Dennis A. Hesselink, Carla C. Baan

**Affiliations:** Rotterdam Transplant Group, Department of Internal Medicine, Nephrology and Transplantation, Erasmus MC, Erasmus University Medical Center, Rotterdam, Netherlands

**Keywords:** solid organ transplantation, allograft rejection, endothelial cells, organoids, 3D *in vitro* models, organ-on-a-chip

## Abstract

The endothelium plays a key role in acute and chronic rejection of solid organ transplants. During both processes the endothelium is damaged often with major consequences for organ function. Also, endothelial cells (EC) have antigen-presenting properties and can in this manner initiate and enhance alloreactive immune responses. For decades, knowledge about these roles of EC have been obtained by studying both *in vitro* and *in vivo* models. These experimental models poorly imitate the immune response in patients and might explain why the discovery and development of agents that control EC responses is hampered. In recent years, various innovative human 3D *in vitro* models mimicking *in vivo* organ structure and function have been developed. These models will extend the knowledge about the diverse roles of EC in allograft rejection and will hopefully lead to discoveries of new targets that are involved in the interactions between the donor organ EC and the recipient's immune system. Moreover, these models can be used to gain a better insight in the mode of action of the currently prescribed immunosuppression and will enhance the development of novel therapeutics aiming to reduce allograft rejection and prolong graft survival.

## Introduction

The endothelium has an important role in transplantation. First, a well-functioning endothelium is crucial for the health of a transplant as it regulates oxygen and nutrient provision to that organ. Second, endothelial cells (EC) play an active role in allograft rejection (1–3).

The endothelium of the transplant is the first contact site for the recipient's immune system with donor cells. This barrier is important as it regulates the flux of immune cells into and out of the transplanted organ, which is essential for protecting the allograft from pathogens (2, 3). However, in the setting of solid organ transplantation, the endothelial barrier also facilitates influx of alloreactive immune cells that can damage the allograft and subsequently lead to allograft rejection (1, 4, 5). Transendothelial migration of recipient immune cells consists of several steps in which immune cells are attracted, roll along, adhere, and eventually migrate through the endothelium (2, 6). In this cascade of events the expression of specific membrane molecules—selectins, integrins, and cytokine-induced adhesion molecules (CAMs)—on both EC and immune cells play a key role. Upon activation, EC increase the expression of these membrane molecules, along with the release of several pro-inflammatory cytokines and chemokines and the loosening of intercellular vascular endothelial cadherin (VE-cadherin) junctions (4, 5, 7, 8). In organ transplantation, several pathways can lead to such EC activation resulting in enhanced influx of (alloreactive) immune cells.

EC activation takes place during the transplantation procedure, when EC of the allograft are affected by ischemia and reperfusion injury (IRI). The temporary absence of circulation causes hypoxia and leads to activation and injury of the donor endothelium even before explantation of the organ (9–11). Upon reperfusion of the donor organ in the recipient, reactive oxygen species (ROS) are produced causing a second wave of EC injury. This results in apoptosis, necrosis and autophagy of EC. Moreover, EC injury and activation leads to a general immune response and chemotaxis of immune cells (12, 13).

EC activation is also seen in different types of allograft rejection, in which EC can be both target and stimulator of the rejection response, i.e., in both cellular and humoral rejection (1, 5, 14, 15). The earliest rejection event after transplantation is known as hyperacute antibody mediated rejection (ABMR), in which preformed donor-specific antibodies (DSA) recognize human leukocyte antigen (HLA) or ABO antigens on EC. Resting EC express HLA class I molecules, but activated EC highly increase the expression of both HLA class I and HLA class II molecules. Therefore, during IRI, preformed anti-HLA DSA easily recognize and subsequently damage the EC and graft. Other than the IRI pathway, DSA binding itself also triggers the activation of EC (14, 16, 17). However, such hyperacute ABMR events are barely seen nowadays, mainly due to improved pre-transplantation screenings.

At later stages after transplantation acute ABMR can develop in which *de novo* anti-HLA DSA or non-HLA EC targeted antibodies are involved. These antibodies can cause activation and destruction of the donor endothelium in which either complement dependent cellular cytotoxicity (CDCC) or antibody-dependent cell-mediated cytotoxicity (ADCC) occurs (14, 15, 18, 19).

Also, cytotoxic lymphocytes (CTL) can recognize donor HLA class I and kill donor EC, which is typically seen in T cell mediated rejection (TCMR) (2, 18, 20). These CTL can be activated by antigen presentation of both donor and recipient APCs within secondary lymphoid organs (18).

Moreover, like professional APC, the endothelium itself is also capable of initiating alloreactive T cell responses. The mechanisms through which this occurs are via enhanced expression of donor HLA class I and II on the EC surface, provision of costimulatory signals to lymphocytes, and cytokine production (21, 22). Through direct and indirect pathways EC can activate CD4 or CD8 memory T cells, which can subsequently result in TCMR (2, 18, 23).

More recently, another form of acute allograft rejection has been recognized, in which microvascular inflammation is not caused by classical ABMR or TCMR pathways, but in which NK cells play a dominant role. HLA-I expression on EC is found to directly activate recipient NK cells, which subsequently leads to EC damage (24).

In addition to acute rejection events, it is hypothesized that subclinical EC activation is involved in the development of chronic allograft rejection. However, this mechanism is less well-understood (14, 23, 25).

EC of the transplant receive the first blow during and after transplantation. However, EC possess a great regenerative capacity (26, 27). EC of donor origin have the capacity to proliferate and replace lost or injured cells. In addition to donor EC, also recipient EC can replace the destructed donor EC to restore the continuum of the vessel wall, leading to EC chimerism. It is hypothesized that EC chimerism might lead to reduced immunogenicity of the endothelium (28–34).

It is clear that EC play a key role in organ transplantation, but research gaps are still present, including (i) the precise role of non-HLA endothelial targeted alloantibodies [i.e., MHC class I related chain A (MICA) and anti-endothelial cell antibodies (AECA)] and (ii) the incidence and degree of EC chimerism and relation with immunogenicity.

Much of our current knowledge on the role of EC in alloreactivity comes from *in vitro* and *in vivo* research models. In this review we discuss the value and limitations of these research models and introduce novel innovative research models that may enable us to profoundly explore the interaction of immune cells with EC in allograft rejection. This will help us to find new targets to protect the graft EC from damage as well as to explore novel treatments to reduce allograft rejection and prolong graft survival.

## Conventional Models for Studying the Role of Endothelial Cells in Allograft Rejection

### Conventional Models

The most-used *in vitro* experimental setup to study the role of EC in allograft rejection is the static 2D co-culture of human umbilical vein endothelial cells (HUVEC) with HLA-mismatched immune cells. Functional analyses are performed by activation, proliferation, transmigration, suppression, and cytotoxicity assays. In these models, cell interactions are studied by flow cytometry, immunofluorescence microscopy, multiplex protein assays, and gene expression analyses (20–22, 35–60). Several agents that specifically block HLA molecules, costimulatory molecules or cytokines have been studied with these methods to investigate their effect on activation, proliferation, and transmigration of immune cells (21, 22, 35, 36, 38, 39, 44, 48, 50, 60). Also, the effect of immunosuppressive drugs on EC and their interaction with immune cells have been examined with these experimental models (22, 45, 52, 61–67).

Animal models have been used to study the role of EC in allograft rejection in a physiological setting. Mostly rodent models are used to transplant organs between major histocompatibility complex (MHC)-mismatched rodent strains. Subsequently, EC—immune cell interactions are studied in blood samples and harvested organs (e.g., the transplanted organ and the secondary lymphoid organs) (21, 29, 40, 42, 68–77). Also, the effect of several immunosuppressive drugs on EC have been investigated within these models, for example to investigate if these drugs can protect EC from IRI injury and reduce EC activation and immune cell infiltration (29, 69, 71, 75).

### Limitations of Conventional Models

Despite the breakthroughs that have been achieved with conventional experimental models, novel models that better mimic the human EC—immune cell spatiotemporal interaction in the allograft are needed in the search for effective therapies as conventional models have some limitations: Although easy to work with, HUVEC are not *per se* the best cell type to study allograft rejection *in vitro* as EC of different origin are very heterogeneous and differ in their shape, cell-cell connections, membrane molecules and permeability. Especially macrovascular EC, like HUVEC, and microvascular EC are highly divergent. Among EC of different organs—and even within an organ- many differences exist as well (8, 78–83). Also, EC from patients may be affected by their condition (for example aging, comorbidity, and drugs) and therefore behave differently than EC derived from healthy controls. For specific research aims, this issue can be bypassed by using patient derived EC. For example, an EC crossmatch model to study ABMR has been established with the use of transplant donor derived EC and corresponding recipient sera (59, 84).

Furthermore, 2D monolayer cultures lack spatial interaction with other neighboring cell types such as pericytes and smooth muscle cells (SMC) which should also be included in the experimental setup (85). Moreover, the EC glycocalyx protects EC from interaction with immune cells, but the glycocalyx is not represented well in conventional static 2D models (86, 87).

Another issue is that static 2D culture conditions lack physiological perfusion and vessel resistance. The importance of mimicking a physiological perfusion of EC is emphasized by the fact that the level of shear stress along with the flow pattern (laminar or disturbed) highly influences EC morphology, migration, proliferation, gene expression, and permeability (88, 89).

Lack of flow and lack of multicellular spatial interaction are not an issue in animal models. However, limiting factors of animal models are the huge genetic, immunological, and pharmacokinetic differences between animals and humans (37, 90–96). Also, many differences between human and animal surgical transplantation procedures exist (97–100). Therefore, results from these animal models cannot be fully translated to human solid organ transplantation. Also, the ethical dilemmas about animal models are growing worldwide and ethical approval for animal studies has become much stricter in most countries.

Recently, novel innovative models for studying tissue and immune interactions *in vitro* have been developed. These models may overcome several of the limitations of conventional models and could also be useful for studying the role of EC in allograft rejection. Below an overview of the most promising models is provided. Table 1 summarizes advantages and limitations of both conventional and novel models.

Table 1Advantages and limitations among conventional and innovative research models.

**Table:** 

	**Conventional models**	
	**Static 2D *in vitro* models**	**Animal models**
**Advantages**	• Easy to perform • Well-established	• Physiological aspects • Allows transplant procedures
**Limitations**	• Cell lines differ from patient derived cells • Lack of glycocalyx • 2D condition and lack of flow highly influences EC morphology and function • Lack of interaction with other cell types	• Genetic, immunological, and pharmacokinetic differences compared to humans • Surgical differences and difficulties compared to human transplantation • Emerging ethical concerns • Time-consuming

**Table:** 

	**Innovative models**			
	**2D dynamic models**	**Humanized rodent models**	**Organ-on-a-chip models**	**Organoids**
**Advantages**	• Continuous flow of medium, supply of O_2_ and growth factors, and removal of waste	• Allograft from human origin • Study interaction of human EC and human PBMC	• Incorporation of diverse cell types • Various methods to create 3D vascularization mimicking *in vivo* perfusion • Possibility to connect multiple organs on a chip	• High-complex organ-like structures that closely resemble *in vivo* organs • Possibility to connect multiple organoids on a chip
**Limitations**	• 2D condition influences EC morphology and function • No organ-like architecture	• No solid organ model available • Translation from skin and aorta models to solid organs is limited • Requires high surgical skills	• Medium-high complexity (to perform) • Organ architecture is still simplistic • Time-consuming	• High complexity (to perform) • Vascularization is not well-developed yet • Tumor/teratoma formation • Immature (i.e., fetal) organs • Time-consuming

## Advanced Experimental Models to Study the Role of Endothelial Cells in Allograft Rejection

### 2D Microfluidic Culture Systems

**Figure 1:**
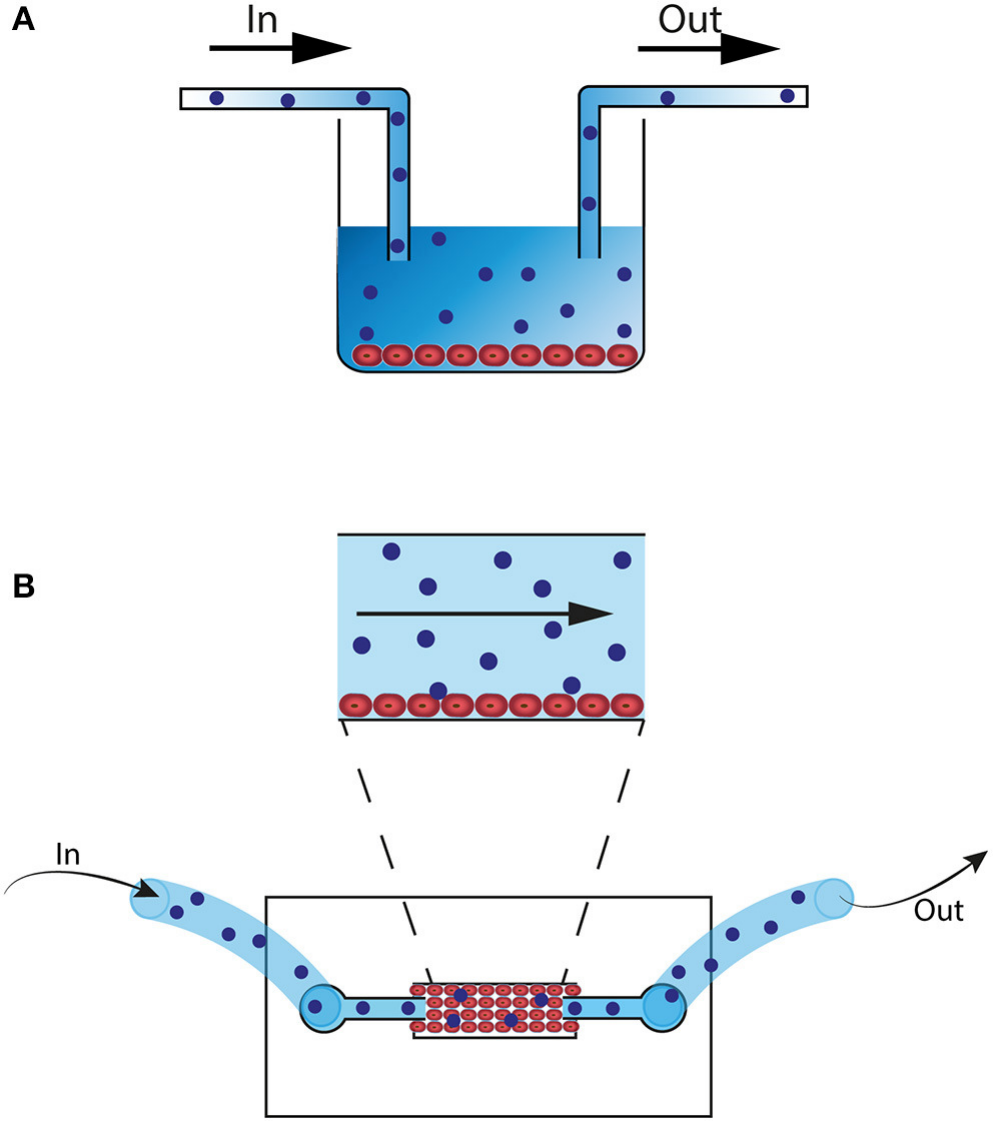
2D microfluidic *in vitro* models. **(A)** Culture well with HUVEC or organ-specific EC (red). The well is connected with a pump (not shown) and flow tubes. Alloreactive lymphocytes (blue) are perfused through the well. **(B)** 2D Microfluidic chip that can be connected to a pump (not shown) and flow tubes. EC (red) are perfused through the system and cells attach to the bottom of the chip. Next, alloreactive lymphocytes (blue) are perfused through the system.

When using a 2D co-culture model with an endothelial cell line and immune cells, a few adjustments can highly improve the system. A great step forward toward more physiological conditions can be made when changing from static to dynamic co-culture to mimic vessel perfusion. Conventional culture plates or microfluidic chips can be connected to peristaltic or pulsatile pumps to create a continuous flow of cell medium, serum or plasma (Figure 1) (101–103). These dynamic flow systems have already been successfully used for studying endothelial and immune cell interactions (101, 104, 105). In addition to simulating physiological perfusion, such systems allow studying the effects of abnormal flow, for example to investigate the effect of discontinued flow which occurs in organs during the surgical transplant procedure (102, 103, 106). Other important steps to improve the 2D co-culture system are choosing the microvascular endothelial cell line of the organ of interest and including neighboring cell types in the system, like pericytes or SMC, through a 2D two-layer design (107).

### Humanized Rodent Allograft Models

Humanized rodent models can be a great improvement of the more conventional rodent models as these humanized models can overcome some of the translational problems that come with differences among species. The definition of a humanized rodent allograft model is that human immune cells are injected and human tissue is transplanted in immunodeficient mice (108–110). For studying the role of EC in transplantation it is important that both EC and immune cells are of human origin to closely resemble human transplantation conditions. With this assumption, a few interesting models can be mentioned; First, the transplantation of human skin onto immunodeficient mice and subsequent injection of human peripheral blood mononuclear cells (PBMC) (21, 70, 71). This model is suitable for studying human EC and human immune cells in an *in vivo* condition and especially the administration of immunosuppressive drugs and its effect on the EC—immune cell interaction would be intriguing to study within this model. However, an important limiting factor of this model is that vascularization of skin transplantation is incomparable with solid organ transplants, as skin transplant vascularization is approximately achieved after 48 hours and completed after 8 up to 21 days (97, 98, 111). Also, vascularization in skin allografts is dominated by recipient neoangiogenesis and regression of donor vasculature, in contrast to human solid organ transplants in which the donor vasculature remains largely intact (111). Another interesting model is the transplantation of human aorta segments into immunodeficient mice and subsequent injection of human PBMC (68). As described above, microvascular EC of solid organs greatly vary from macrovascular EC of an aorta segment. Likewise, the aorta model does not allow for studying the interaction and transmigration of immune cells through microvascular graft EC. Combined transplantation of human skin and human coronary artery in immunodeficient mice has also been performed. In this study subsets of immune cells have been injected to study the effect of specific immune cell populations on the allograft (112).

### 3D Culture Systems: Organs on a Chip and Organoids (on a Chip)

#### Organ on a Chip

**Figure 2:**
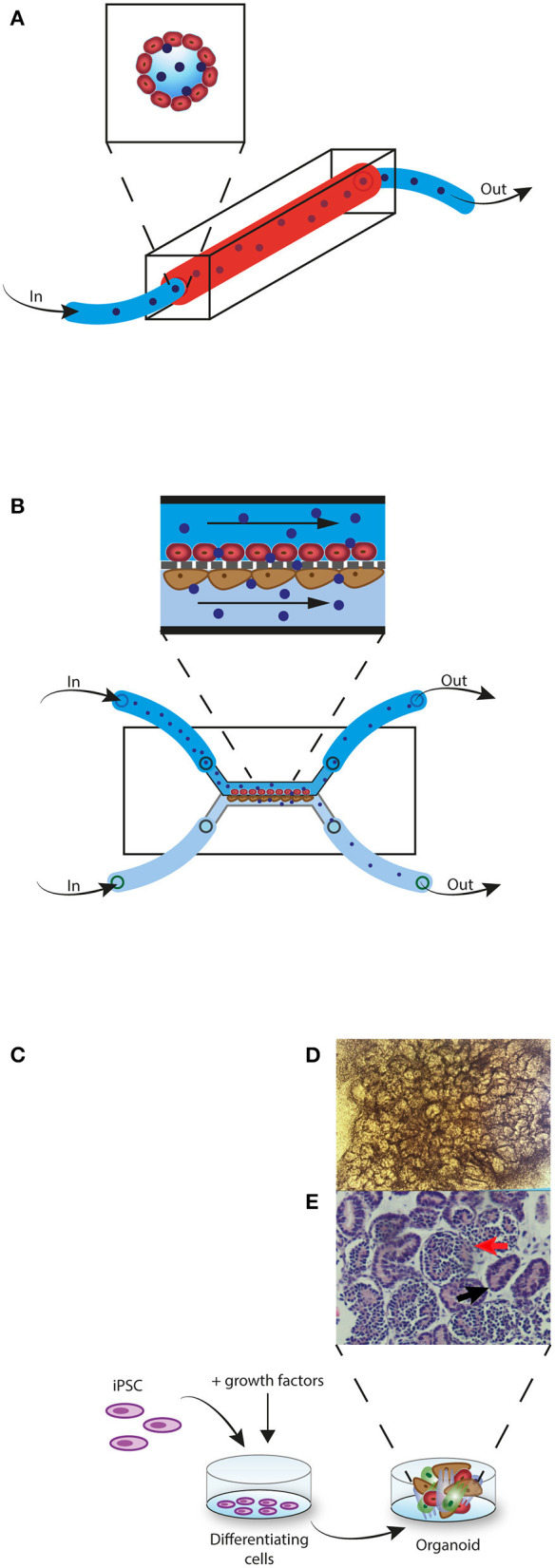
3D *in vitro* models. **(A)** Vessel-on-a-chip model. EC (red) are perfused through a PDMS mold channel. When confluent, alloreactive lymphocytes (blue) are perfused through the vessel. **(B)** Glomerulus-on-a-chip model. EC (red) are perfused through the upper channel and podocytes (brown) are perfused through the lower channel. The two cell layers are separated by a membrane that imitates the glomerular basement membrane. Next, alloreactive lymphocytes (blue) are perfused through the upper channel. Non-migrated lymphocytes are collected from the upper channel and migrated lymphocytes are collected in the lower channel. **(C)** Kidney-organoid model. iPSC are induced with growth factors to differentiate into a 3D multicellular structure. **(D)** Complex structure formation can be monitored with light microscopy. **(E)** Histological H&E staining of a kidney-organoid shows tubular (black arrow) and glomerular structures (red arrow).

Another promising transition is the switch from 2D to 3D models. In parallel with the upcoming 3R movement—i.e., reduction, refinement, and replacement of animal models—the bioengineering industry has made big steps forward in producing 3D *in vitro* models that closely resemble organ function, the so called organ-on-a-chip models. The most developed “organ” on a chip is the vessel-on-a-chip (113, 114). In this model a silicon-based—Polydimethylsiloxane (PDMS)—mold is used in which channels are artificially created with a biopsy punch. The channels are then perfused with EC that will grow into 3D vessel-like structures (Figure 2A) (113, 115–118). PDMS-based molds have limitations however, as this material can absorb certain small molecules, including oxygen and drugs, leading to distorted results (119, 120). Coating the PDMS membrane with a lipid-based solution, before adding the EC, has overcome this problem (119). Recently, the introduction of hydrogel-based molds, the addition of surrounding cells (pericytes or SMC) and the use of the 3D printing technique has resulted in even more realistic vessels in which the effect of shear stress, neighboring cells, and extracellular matrix (ECM) can be studied (121–126). Even naturally occurring neo-angiogenesis and sprouting resulting in self-structured vessels has been made possible (124, 127). These self-structured vessels however, are yet in an early stage as perfusion in self-formed vessels and branches cannot always be assured. Depending on the research purpose either the guided vessel formation or the self-structured vessels may be the best model to choose. These vessel-on-a-chip models can be perfused with cell medium, drugs, serum, plasma or even whole blood to study for example, thrombosis and drug pharmacokinetics and -dynamics (Figure 2A) (114, 115, 123, 128–130). Oxygen can also be added to the perfusion fluid (131). One research group designed a vessel-on-a-chip system as a model for xenotransplantation. Microfluidic chips containing microchannels were constructed with the use of PDMS and mold needles. The channels were perfused with porcine aortic endothelial cells (PAEC) and confluency was confirmed with confocal microscopy. Next, PAEC vessels were perfused with human serum. Confocal microscopy and protein assays were used to investigate the effect of the human serum perfusion on PAEC and complement activation (123). 3D vessel-on-a-chip models would also be a great step forward in the research to the role of EC in allograft rejection for example to study (i) the effect of induced hypoxia and reperfusion on EC (ii) the interaction of EC and alloreactive immune cells (iii) the effect of immunosuppressive drugs on EC activation and on transmigrating immune cells, (iv) EC chimerism.

Other than vessels, many organ-on-a-chip models have been generated, including kidney, liver, lung, heart, brain, skin, gut, and lymph nodes (130, 132). Pre-differentiated cells—cell lines or human pluripotent stem cell (hPSC) derived cells—are used in these models. In some of these organ-on-a-chips an endothelial barrier is incorporated in the model and is therefore interesting for studying the role of EC in allograft rejection. For example, a glomerulus on a chip was generated with hPSC-derived podocytes, glomerular microvascular endothelial cells (GMEC) and a glomerular basement membrane (GBM)-like structure (133). Figure 2B shows an example of a glomerulus-on-a-chip model. Also, a 3D vascularized proximal tubulus chip has been established in which a tube formed by proximal tubular epithelial cells (PTEC) and a second tube formed by GMEC are separated by an ECM-like membrane (134). Both glomerulus- and tubulus-on a chip systems have been able to simulate glomerular filtration (133–136). These models would be very interesting in studying the role of EC in transplantation. For example to study (i) how alloreactive immune cells that transmigrate through the endothelium have an effect on the tubular cells or podocytes and by which mechanisms (ii) to discover new drug targets that could interfere with this process (iii) to investigate the effect of currently used immunosuppressive drugs (iv) or to investigate if alloreactive immune cells have a different effect on glomerular EC compared to tubular EC.

The choice for a specific EC cell source in an organ-on-a-chip model comes with certain advantages as well as limitations. Primary EC most closely resemble *in vivo* conditions, but are limited in culture time as they lose phenotype and functional characteristics after a few passages. Also inter-individual variation occurs within primary cells (137). Immortalized EC cell lines can, in contrast to primary cells, be kept in culture for long term and do not have the issue with between-donor variation. These EC cell lines, however, generally do not resemble well *in vivo* conditions (137, 138). Circulating progenitor endothelial cells (EPC) have occasionally been used for *in vitro* differentiation into mature EC, but are limited in their use due to the low yield and the controversy about their identification (139, 140). hPSC have been used for cell differentiation of many different cell lineages, including EC. The ability to expand these stem cells for long term is a great advantage, but a limitation is their immaturity and their phenotypic instability after differentiation (137, 141, 142). Moreover, developed differentiation protocols are generated with the use of a few stem cell lines and application by researchers with other stem cell lines often leads to reproducibility problems.

A new concept of organs-on-a-chip is the connection of individual organ-chips to create a body-on-a-chip (143–145). Especially for drug development and for *in vitro* modeling of diseases that affect multiple organs, body-on-a-chips would be very useful. The current challenge for this model is to enhance vascularization and innervation of all the organs in the system (143). Also, defining the proportional size of each organ in the system and facilitate maturation of all organs at the same time are remaining challenges (143, 146). Connecting only a few organs instead of creating a whole-body-chip might be sufficient for some research purposes and could reduce some of the described challenges. For studying the role of EC in allograft rejection, it would be interesting to connect a kidney-, liver- or heart-on-a-chip with a lymph-node-on-a-chip and generate vascularization and lymphatic drainage. In cancer research a vascularized and lymphatic drained tumor-on-a-chip has already been developed (147). With such a system, in which the organ-on-chip and lymph-node-on-chip are alloreactive to each other, it would be possible for example, to study the difference between alloreactive T cells that are either activated by the endothelium or activated by DCs in the lymph node and subsequently, if the effect between these two populations within the organ is distinctive. Also, it is well-known that upon damage, EC can detach and circulate through the blood (148, 149). If these circulating endothelial cells (CEC) are also capable of antigen presentation in the circulation or within the lymph node is unknown, but it may be possible to investigate this with such a model as well.

#### Organoids (on a Chip)

A more complex model of an organ *in vitro* has been achieved by the establishment of the organoid technology by which hPSC- including induced pluripotent stem cells (iPSC) and embryonic stem cells (ESC)- or adult stem cells (ASC) can be induced to grow into multicellular self-organized 3D organ-like structures (150). Organoids are compared to organs-on-a-chip a more advanced model of true organs as organoids have a higher complexity of (organ) structure and function. Specific signaling molecules of the embryonic development are needed for the differentiation of stem cells into organ-like structures, corresponding to the organ of interest (150–152). Depending on whether hPSC or ASC are used, organoids resemble a fetal-like organ or a matured adult-like organ (151, 153, 154). Other important differences between the various sources of stem cells are: (i) ASC are multipotent and can therefore not be differentiated in all cell types, in contrast to the pluripotent hPSC. (ii) ESC availability is limited as these cells are derived from human embryos, which also gives rise to ethical concerns. (iii) For modeling of genetic diseases the use of iPSC is specifically interesting as iPSC can be derived from patients with the disease of interest (137, 150, 152).

Various types of organoids have been developed, including brain, gut, liver, pancreas, lung, heart, and kidney organoids (150, 152). Figures 2C–E shows an example of kidney organoid formation with iPSC. Diverse platforms for the generation of organoids are used, ranging from culture on a solid ECM base, culturing in suspension with or without ECM proteins, or culturing with an air-liquid interface (151).

For the use of organoids as a platform to study the role of EC in allograft rejection, it is important whether or not EC are present in the organoids. It has been shown that EC can be represented within organoids, but spontaneous development of vascular networks *in vitro* is absent (155–159). Vascularization has, however, been achieved by the transfer of *in vitro* grown organoids into immunodeficient mice (152, 157, 158, 160). As animal models are unwanted, it has been suggested that combining the organ-on-a-chip technology with organoids could be an attractive approach to realize *in vitro* vascularized organoids (161–163). Several attempts to reach this goal have been performed. One study generated kidney organoids on a scaffold in a 3D microfluidic system. This resulted in the outgrowth of organoid-derived EC into a perfusable vascular network within the organoids (164). However, the success of this vascularization and perfusion has been questioned by other researchers (165). Also, vascularization of brain organoids *in vitro* has been reported but the possibility of *in vitro* perfusion of the organoid-infiltrating vessels has not been investigated (159). Another reported method to perfuse organoids is the use of 3D printing to create a channel network within cardiac organoids. The channels can be connected to a pump and perfused with oxygenated cell medium. However, the perfusion of HUVEC in the channels to mimic blood vessels resulted in incomplete coverage of the channels (166). Liver organoids with incorporated EC have been generated by co-culturing hPSC-derived pre-hepatic cells together with EC. Although *in vitro* vascular networks did not occur, hepatic function of the organoids improved by the presence of EC (156–158). This is an intriguing finding and suggests that studying EC function in organoids is worthwhile despite the lack of real vascular networks. Therefore, this model allows the study of the immunogenicity of EC in organoids against alloreactive immune cells by co-culturing alloreactive immune cells with the EC-containing organoids.

Ideally, *in vitro* vascularized organoid models to study the role of EC in allograft rejection should include organ specific EC. For such models, EC originating from the organoid itself, organ specific primary EC or organ specific EC differentiated from hPSC could be used (159, 164). Organoid vascularization with non-organ specific EC, like HUVEC, may not resemble organ function properly (156–158, 166). For example, glomerular EC have specific characteristics to support filtration which can not be substituted by HUVEC (8). Technical challenges however, could hamper the practical feasibility of such elegant models that include organ specific EC.

Some other limitations of the organoid methodology makes their use as a research model a challenge. First, remaining undifferentiated hPSC within the organoids can transform into tumors/teratomas (167, 168). Second, some organoids, including brain and liver organoids, can be cultured for a long term (up to several months), but other organoids, including kidney organoids, cannot be maintained in culture for more than a few weeks. This could make certain research aims difficult to fulfill (152, 169). Third, the immaturity of hPSC derived organoids could also be a limiting factor (151, 153). Finally, organoids are highly heterogeneous and variation in organoid quality is seen between experiments, between hPSC lines, and between cell batches (169–171).

Despite of these limitations, *in vitro* perfusable vascularized organoids are a promising research platform to investigate the role of EC in allograft rejection, but optimization of vascularization is needed first. Also, in accordance to the body-on-a-chip development, investigation of connecting multiple organoids-on-a-chip is in progress as well. Such systemic models with various organs represented is a hopeful model for many research areas, including drug development, and transplantation research (162, 172).

### Isolation and Characterization of EC for Use in Advanced Experimental Models

Various sources of EC have been used in the above described advanced experimental models. The degree of complexity of isolation and characterization methods differs between various EC sources and may therefore influence the choice for a certain EC source to be used in an advanced experimental model. Primary EC can be commercially purchased or isolated from explanted tissue of biopsy material. Especially in models with disease specific EC, isolation from patient derived tissue is preferred and one should consider which method is most appropriate.

Isolation of conventionally used HUVEC is relatively easy compared to microvascular EC (173, 174). The inner lining of umbilical veins is enzymatically digested with collagenase treatment or mechanically scraped and results in a substantial yield of EC and relatively low contamination with other cell types (173, 175). Isolation of microvascular EC from solid organs requires enzymatic digestion of tissue resulting in a lower degree of EC purity and therefore subsequential EC purification is needed (174, 176, 177). Within an organ, different types of microvascular EC can be present and it may be useful to separate those depending on the research question (8, 178). For example, within the kidney, separation of glomerular and tubulo-interstitial EC can be performed by collagenase treatment of renal cortex and next passing the obtained cell suspension through different pore size sieves, which will separate glomerular EC from tubulointerstitial EC (174, 179). After enzymatic digestion, the obtained cell suspension is often added to a gelatin-coated culture flask to allow for EC adhesion, expansion and purification. Non-adherent cell types are removed upon the first cell culture medium refreshment (173, 175–177, 179). EC can be visually recognized by their typical cobblestone appearance, which allows for EC purification by manual weeding (173–175). Another method that can be used to remove fibroblasts and mesenchymal cell contamination is with a DiI-conjugated acetylated low-density lipoproteins (DiI-AC-LDL) assay. EC take up DiI-AC-LDL whereas fibroblasts and mesenchymal cells do not. After incubation of cell cultures with DiI-AC-LDL, uptake assessment and EC purification can be performed by fluorescence microscopy along with manual weeding or by flow cytometric cell sorting (176, 179). A third EC purification method is based on positive or negative cell selection with the use of magnetic beads, for which most often anti-platelet endothelial cell adhesion molecule (PECAM) beads, anti-von Willebrand factor (vWF) beads or Ulex europaeus agglutinin-1 (UEA-1) beads are used. This magnetic purification step can be performed immediately after enzymatic tissue digestion before the cells are added to a culture flask or the purification can be done or repeated after a certain period of cell culture (174, 176, 177, 179). The obtained primary EC can be used in an experimental model or can be transformed into immortalized EC lines by viral transduction and subcloning. Immortalized EC should then be assessed for the persistence of EC characteristics, as the immortalization process often leads to diminished expression of EC membrane molecules (180–182).

EC can also be acquired through differentiation of hPSC. The protocol for EC differentiation most often includes bone morphogenic protein-4 (BMP4), fibroblast growth factor 2 (FGF2) and vascular endothelial growth factor (VEGF) (141, 142, 159, 183, 184). During the differentiation process, it may be required to perform a purification step based on the expression of EC specific markers, for example PECAM, VE-cadherin, kinase insert domain receptor (KDR) and neuropilin-1 (NPR1) are used (141, 142, 184). However, it has been reported that the generation of a high purity EC population is possible without cell sorting (159, 183). Successful EC differentiation can subsequently be confirmed by assessment of general EC characteristics, including cobblestone appearance, DiI-AC-LDL uptake and expression of PECAM, VE-cadherin, and vWf (141, 142, 183).

## Conclusion

We have provided an overview of innovative research models that will greatly improve the study of EC in solid organ transplantation. The first step in improving the relevance of research models is the transition from 2D static to 2D dynamic culture models and from conventional animal models to humanized rodent models. Further progress can be made with the implementation of 3D *in vitro* research models. Substantial translational limitations of both 2D models and animal models can be overcome with the use of these models. Implementation of the 3D models will therefore lead to increased knowledge about the role of EC in allograft rejection and to the discovery of new targets for drug development. Moreover, new insights about the effect of currently used immunosuppressive drugs on the EC- immune system interaction can be obtained.

Although some 3D models are still in early developmental stage, their upcoming use in various research fields will soon redress current restraints. Other research areas focusing on EC will also benefit by using these models, for example the study of EC in cardiovascular diseases. Further, the proposed models are likewise feasible for wider applications, including transplantation research in general and other research areas (e.g., immunology, cancer, and pharmacology research).

## Author Contributions

DP writing original draft. DP, MH, DH, and CB editing. MH, DH, and CB supervision. All authors contributed to the article and approved the submitted version.

## Conflict of Interest

The authors declare that the research was conducted in the absence of any commercial or financial relationships that could be construed as a potential conflict of interest.
